# Insufficiency of DNA repair enzyme ATM promotes naive CD4 T-cell loss in chronic hepatitis C virus infection

**DOI:** 10.1038/s41421-018-0015-4

**Published:** 2018-04-10

**Authors:** Juan Zhao, Xindi Dang, Peixin Zhang, Lam Nhat Nguyen, Dechao Cao, Lin Wang, Xiaoyuan Wu, Zheng D Morrison, Ying Zhang, Zhansheng Jia, Qian Xie, Ling Wang, Shunbin Ning, Mohamed EL Gazzar, Jonathan P Moorman, Zhi Q Yao

**Affiliations:** 1 Center of Excellence in Inflammation, Infectious Disease and Immunity, James H Quillen College of Medicine, East Tennessee State University, Johnson City, TN 37614 USA; 2 Department of Internal Medicine, Division of Infectious, Inflammatory and Immunologic Diseases, Quillen College of Medicine, ETSU, Johnson City, TN 37614 USA; 3 Department of Infectious Diseases, Tangdu Hospital, the Fourth Military Medical University, Xi’an 710038, China; 4 Department of Biomedical Science, James H Quillen College of Medicine, East Tennessee State University, Johnson City, TN 37614 USA; 5 Department of Veterans Affairs, Hepatitis (HCV/HIV) Program, James H Quillen VA Medical Center, Johnson City, TN 37614 USA

## Abstract

T cells have a crucial role in viral clearance and vaccine response; however, the mechanisms regulating their responses to viral infections or vaccinations remain elusive. In this study, we investigated T-cell homeostasis, apoptosis, DNA damage, and repair machineries in a large cohort of subjects with hepatitis C virus (HCV) infection. We found that naive CD4 T cells in chronically HCV-infected individuals (HCV T cells) were significantly reduced compared with age-matched healthy subjects. In addition, HCV T cells were prone to apoptosis and DNA damage, as evidenced by increased 8-oxoguanine expression and γH2AX/53BP1-formed DNA damage foci—hallmarks of DNA damage responses. Mechanistically, the activation of DNA repair enzyme ataxia telangiectasia mutated (ATM) was dampened in HCV T cells. ATM activation was also diminished in healthy T cells exposed to ATM inhibitor or to HCV (core protein) that inhibits the phosphoinositide 3 kinase pathway, mimicking the biological effects in HCV T cells. Importantly, ectopic expression of ATM was sufficient to repair the DNA damage, survival deficit, and cell dysfunctions in HCV T cells. Our results demonstrate that insufficient DNA repair enzyme ATM leads to increased DNA damage and renders HCV T cells prone to apoptotic death, which contribute to the loss of naive T cells in HCV infection. Our study reveals a novel mechanism for T-cell dysregulation and viral persistence, providing a new strategy to improve immunotherapy and vaccine responses against human viral diseases.

## Introduction

Hepatitis C virus (HCV) is a blood-born pathogen characterized by a high rate (>80%) of chronic infection, which can progress to liver cirrhosis and hepatocellular carcinoma—a leading cause for liver transplantation^1^. Notably, HCV has evolved numerous strategies to evade host immunity and harness virus persistence^1^, providing an excellent model to study the mechanisms of virus-mediated host immune dysfunction in humans. We and others have previously reported that patients with chronic HCV infection exhibit premature T-cell aging, as demonstrated by overexpression of aging markers and telomere attrition—indicating excessive proliferative turnover or inadequate telomeric maintenance^2–6^. However, the molecular mechanisms that control T-cell homeostasis and virus persistence in humans remain unclear.

T-cell homeostasis is tightly controlled, requiring a fine balance between influx of newly generated T cells from the thymus and efflux by consumption via T-cell apoptosis, and self-replication within the existing pools of T lymphocytes^7, 8^. With deficient thymic influx in aging adults, the immune system responds to in vivo and in vitro challenges by expanding existing T cells, leading to increased proliferative turnover, telomere attrition, and cell apoptosis^7, 8^. We hypothesize that premature T-cell aging not only involves virus-specific effector and memory T cells engaging in chronic viral infection, but may also extend to the compartment of naive T cells that are unprimed by antigens. In support of this notion, broad regulatory anomalies, including the markers for T-cell exhaustion and senescence, are found not only expressed on virus-specific T cells, but also on unprimed naive T cells that have not yet engaged in immune responses^2–6, 9–14^. This notion is also supported by the observations that individuals with chronic viral (HCV or HIV) infection often have blunted vaccine responses, suggesting a broad and shared mechanism of immune dysregulation, particularly naive CD4 T-cell dysfunction, and vaccine non-responsiveness in virally infected individuals^2, 3, 15–19^.

Human naive T cells have a relatively long life span (150~160 days) and thus are exposed to a multitude of genotoxic stressors, leading to 1% of a pool of 300 billion T cells to be replaced daily^7, 8^. Notably, naive T cells are typically resistant to death receptor/ligand (Fas/Fas-L)-mediated apoptosis, pointing toward cell-internal signals as apoptosis initiators^20^. One of the internal stressors linked to apoptosis is damaged DNA, which is particularly important in senescent cells that have been chronically exposed to the endogenously generated reactive oxygen species (ROS)^21^. To maintain genomic stability and cell survival, cells continuously recognize and respond to this DNA damage, which will either activate DNA damage checkpoints to arrest cell cycle progression and allow for repair or, if the damaged DNA is beyond repair, undergo apoptosis^22^.

A major sensor of DNA breaks is the MRN complex (MRE11, RAD50, and NBS1), which subsequently recruits the protein kinase ataxia telangiectasia mutated (ATM), an enzyme critically involved in repairing DNA double-strand breaks (DSBs) for cell survival^23, 24^. ATM was originally identified in individuals with ataxia telangiectasis, an autosomal recessive disorder exhibiting progressive ataxia, telangiectasia, immunodeficiency, genome instability, and cancer predisposition^25^. ATM, accompanied by ataxia telangiectasia Rad3-related (ATR) and DNA-dependent protein kinase catalytic subunit c (DNA-PKc), is the pinnacle kinase of the DNA repair signaling cascade, and belongs to the phosphoinositide 3 kinase (PI3K)-related kinase family^26^. Accumulation of DNA-DSBs activates ATM cascades, along with other DNA damage repair machineries, which are important for DNA reprogramming and cell remodeling.

To identify factors that perturb T-cell homeostasis during HCV infection, we investigated the mechanism that controls T-cell survival and DNA damage repair capabilities in primary CD4 T cells. We demonstrate that insufficiency of ATM leads to accumulation of DNA damage, rendering naive CD4 T cells sensitive to apoptosis and T-cell loss, contributing to viral persistence and vaccine non-responsiveness in chronic HCV infection.

## Results

### Naive CD4 T-cell apoptosis and loss in HCV-infected patients

**Fig. 1:**
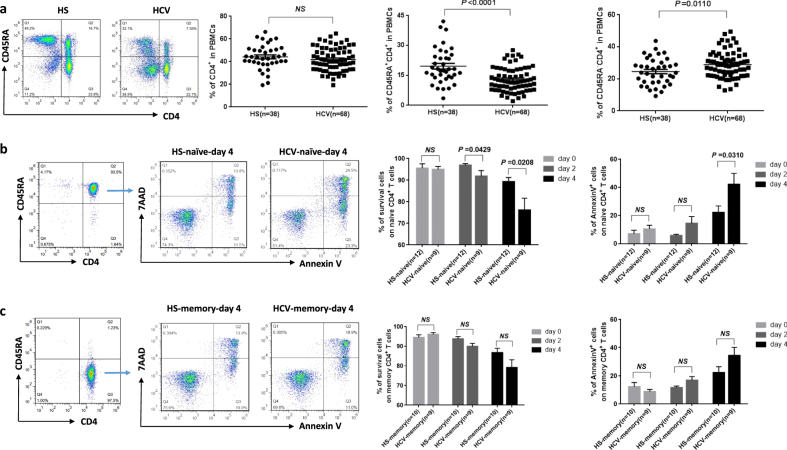
T-cell homeostasis and apoptosis in HCV-infected patients versus age-matched HS. **a** Naive CD4 T-cell loss in HCV patients vs. age-matched HS. PBMCs isolated from HCV-infected patients and HS were analyzed using flow cytometry for T-cell homeostasis. Representative dot plots and summary data of the flow cytometry for the percentages of total CD4^+^, CD45RA^+^CD4^+^ (naive), and CD45RA^−^CD4^+^ (memory) T-cell frequencies in PBMCs from HCV patients and HS are shown. Each symbol represents one particular subject; the mean ± SE and *P* value of the statistical analysis are shown, NS=no significance. **b**, **c** Susceptibility of T cells to spontaneous apoptosis and death in HCV patients vs. HS. Naive and memory CD4 T cells were purified from PBMCs of the subjects, cultured in vitro without stimulation for 0, 2, and 4 days, followed by flow cytometric analysis of AV and 7AAD expression. Representative dot plots for the cell purity, gating strategy, and summary data for the percentages of survival cells as well as apoptotic cells are shown. *n*=number of subjects studied in each group. NS=no significance. P value with significant changes are shown

As an initial approach to identify factors that perturb T-cell homeostasis in HCV infection, we characterized the frequencies of primary T cells and their survival rate or susceptibility to apoptosis in individuals with chronic HCV infection (*n* = 68) versus age-matched healthy subjects (HS) (*n* = 38). We first analyzed total CD4^+^, CD45RA^+^CD4^+^ (naive), and CD45RA^−^CD4^+^ (memory) T-cell frequencies in the peripheral blood mononuclear cells (PBMCs) using flow cytometry. As shown in Fig. 1a (representative dot plots and summary data), while the total CD4 T-cell numbers in PBMCs were slightly lower in HCV patients, the compartment of naive CD4 T cells was significantly reduced (*P* < 0.0001), whereas memory CD4 T cells were expanded in chronically HCV-infected subjects compared to HS. To exclude the possibility that the gated PBMCs may include some CD4-expresing monocytes, we further gated on CD3^+^ T cells, followed by analyzing CD45RA^+^CD3^+^CD4^+^ (naive) and CD45RA^−^CD3^+^CD4^+^ (memory) T-cell populations, which produced similar results; i.e., chronic HCV subjects exhibited a significant contraction of naive T-cell pools and expansion of memory T cells in their peripheral blood (data not shown). Notably, the loss of naive CD4 T cells from chronically HCV-infected subjects did not correlate with HCV genotype, viral load, or hepatic transaminase levels (data not shown). This observation is in line with the previous reports showing a reduced naive CD4 T-cell number—reflecting a state of immune activation and exhaustion in patients with chronic HCV infection^9, 10^.

Apoptosis represents a major mechanism controlling T-cell homeostasis in adults^7^. The vast majority of CD4 T cells in vivo are in a resting state and, accordingly, do not undergo apoptosis. When removed from their natural resources and kept *ex vivo*, human T cells spontaneously and progressively go through the programmed cell death (spontaneous apoptosis). To explore whether apoptosis contributes to the naive T-cell loss in HCV-infected individuals, we purified CD4^+^CD45RO^−^ naive and CD4^+^CD45RA^−^ memory T cells from HCV^+/−^ subjects and cultured the cells without stimulation for 0, 2, 4 days, followed by measuring the Annexin V (Av)/7-Aminoactinomycin D (7AAD) expressions. As shown in Fig. 1b, c, although healthy T cells showed signs of apoptosis/death accrual, an increased T-cell apoptosis and decreased cell survival rate were observed in HCV-infected patients, especially in the naive CD4 T-cell pools. By day 4 in culture without stimulation, ~90% of healthy naive T cells were alive, whereas only 75% of the HCV naive T cells still remained survival. Notably, increased apoptotic propensity (Av expression) was inversely associated with cell survival rate, with HCV naive T cells more prone to spontaneous apoptosis compared with the HS. By day 4 in culture without stimulation, HCV naive T cells exhibited significant apoptosis compared with HS (~40% vs. ~20%, *P* < 0.05), which negatively correlated with the cell numbers present in the peripheral blood from the same individuals (data not shown). These results suggest that apoptotic susceptibility of T cells from HCV-infected subjects may be one mechanism contributing to the disproportionate T-cell loss, whereas lack of thymic influx in aging adults and increases of naive T-cell differentiation into antigen-specific effector and memory T cells during persistent viral infection may be other mechanisms for the different outcomes of the two T-cell subsets.

### DNA damage in naive CD4 T cells from HCV-infected patients

**Fig. 2:**
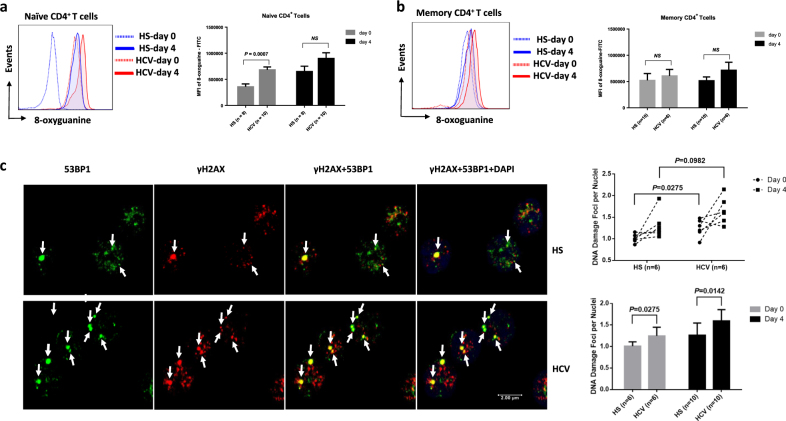
DNA damage in CD4 T cells from HCV-infected patients versus age-matched HS. **a**,** b** 8-oxyguanine expression in CD4 T cells. Nand memory CD4 T cells were purified from PBMCs of HCV-infected patients and HS, cultured in vitro without stimulation for 0–4 days, followed by flow cytometric analysis of 8-oxyguanine expression, a marker for oxidative DNA strand breaks. Representative overlaid histogram and summary data for mean florescence intensity (MFI) of 8-oxyguanine expression in HCV patients and HS are shown. **c** Co-localization of 53BP1 and γH2AX in CD4 T cells. Naive CD4 T cells were isolated from PBMCs of HCV patients and HS, cultured in vitro without stimulation for 0–4 days, followed by confocal microscopic analysis of 53BP1 and γH2AX co-localization in the nuclei, a hallmark of DNA damage foci. 100 cells were counted per subjects, and a total of 10 HCV-infected patients and 10 age-matched HS were studied (six in each group were kinetically analyzed). *P* values of the DNA damage foci per nuclei in the two groups are shown in the summary data

Why naive CD4 T cells are susceptible to apoptosis and loss in virally infected individuals is unclear. Unlike activated or memory T cells, resting naive T cells typically do not express Fas surface receptor (Supplementary Figure S1A). In addition, blocking the extrinsic death pathways by disrupting Fas-Fas ligand, TNFα-TNF receptor, and TRAL–TRAIL receptor interactions in CD4 T cells did not affect cell apoptosis or death rates (data not shown), indicating they are resistant to the exogenous apoptotic pathway-mediated cell death, but sensitive to endogenous oxidative stress, particularly ROS-mediated genotoxicity^20^. To assess endogenous DNA damage as a possible cause of impaired T-cell survival, CD4^+^CD45RO^−^ naive and CD4^+^CD45RA^−^ memory T cells were isolated from HCV-infected patients and age-matched HS, cultured in vitro without stimulation for 0–4 days, followed by DNA integrity analysis by measuring the expression of 8-oxogunine (8-oxoG), a marker for DNA-DSBs that are caused by excessive oxidative stress^21^. As shown in Fig. 2a, b (representative overlaid histogram and summary data of flow cytometry), resting naive CD4 T cells derived from HCV-infected patients had significantly higher expression of 8-oxoG DNA bases compared to HS, indicating an accumulation of DNA lesions during chronic viral infection. After culturing cells without mitogenic or antigenic stimulation, both HCV and HS T cells showed increases in the expression of 8-oxoG. However, the extent of this increase in HCV naive T cells was less than that in HS. This could be attributed to (i) the relatively high baseline 8-oxoG level in HCV naive T cells at day 0, and (ii) the test saturation of 8-oxoG load in T cells under these culture conditions with oxidative DNA stress, which could limit the 8-oxyG differences between HCV and HS T cells at day 4 in culture. In contrast, memory T cells from HCV and HS exhibited an overload of 8-oxoG at baseline. After culturing cells without stimulation, 8-oxoG levels only slightly increased, although HCV memory T cells exhibited marginally higher DNA lesions than HS at baseline (day 0) and 4 days in culture. These data indicate that naive T cells from HCV patients exhibit oxidative DNA-DSBs that remain unrepaired during viral infection.

Following genotoxic insult, histone variant H2AX is recruited to the site of DNA-DSBs and becomes phosphorylated at its C-terminal Ser-139 residue to form the γH2AX complex, which subsequently acts as a docking site for other mediators or adaptor proteins, such as 53BP1, to form microscopically visible nuclear focus (DNA damage foci)—a hallmark of DNA damage response (DDR)^27, 28^. To confirm that DNA damage occurs in T cells during HCV infection, we purified naive and memory CD4 T cells from HCV^+/−^ subjects and compared DNA damage foci by examining the co-localization of γH2AX/53BP1 per nuclei using confocal microscopy. As shown in Fig. 2c (representative imaging and summary data of confocal microscopy), the number of DNA damage foci was significantly higher in the naive CD4 T cells freshly isolated from HCV patients compared with the HS. We also observed an increase in DNA damage foci in memory CD4 T cells derived from HCV patients versus HS (Supplementary Figure S1B). When the cells were cultured without stimulation for 4 days, the DNA damage foci increased, and were significantly higher in the naive CD4 T cells from HCV patients compared with the HS. These results, in conjunction with the changes in T-cell frequency and apoptotic death, suggest that unrepaired DNA damage is associated with T-cell apoptosis and loss in individuals with chronic HCV infection, emphasizing the role of DNA damage repair to secure T-cell survival.

### DNA damage sensing and repairing machineries in naive CD4 T cells from HCV-infected individuals

**Fig. 3:**
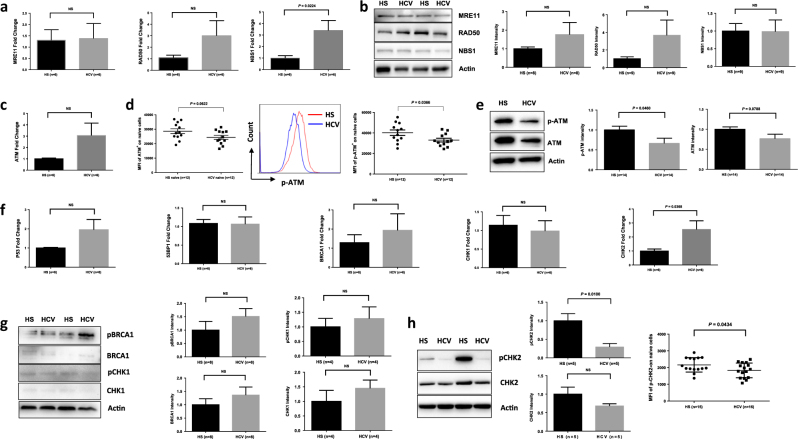
DNA damage repair ATM signaling pathway in T cells of HCV-infected patients versus age-matched HS. **a** DNA damage sensor MRN complex expressions in CD4 T cells. Naive CD4 T cells were isolated from six HCV-infected patients and six age-matched HS, cultured in vitro without stimulation for 4 days, followed by real-time RT-PCR assay for MER11, RAD50, and NBS1 mRNA expression. **b** MRN protein expression detected by western blot in naive CD4 T cells derived from HCV patients vs. HS. Representative imaging and summary data from multiple subjects are shown. **c** ATM mRNA expressions in CD4 T cells. Naive CD4 T cells were isolated from the study subjects as indicated, cultured in vitro without stimulation for 4 days, followed by RT-PCR analysis for ATM mRNA level. **d**,** e** Flow cytometry and western blot analysis for ATM total and phosphorylated protein expression in naive CD4 T cells from HCV patients vs. HS. **f–h** ATM signaling molecule mRNA and protein expressions in CD4 T cells. Naive CD4 T cells were isolated from the study subjects, cultured in vitro without stimulation for 4 days, followed by RT-PCR analysis for P53, BRCA1, CHK1, and CHK2 mRNA level, western blot assay for their total and/or phosphorylated protein levels. pCHK2 expression was confirmed by flow cytometry analysis

Accumulation of DNA-DSBs in CD4 T cells from HCV patients indicates that the DNA damage sensing and repairing machinery is disrupted. Essential components of this machinery include DNA damage sensors, such as MRN complexes (MRE11, RAD50, and NBS1), which recruit and mediate the DNA repair kinase ATM that can phosphorylate several downstream checkpoint proteins (such as p53, BRCA1, Chk1, and Chk2)^22–24^. To investigate the cellular machineries that contribute to the DNA damage repair, we examined mRNA transcripts and protein expressions of these DDR molecules in naive CD4 T cells from HCV-infected patients and HS using real-time RT-PCR and western blotting or flow cytometry. As shown in Fig. 3a, the mRNA levels of MRN complexes showed no difference or higher levels of MER11, RAD50, and NBS1. In parallel, the protein levels of these DNA damage sensors in naive CD4 T cells were also unchanged or slightly higher in HCV compared with HS (Fig. 3b). Intriguingly, although the mRNA level of ATM was higher (Fig. 3c), its protein level was lower; particularly, ATM phosphorylation (pATM) was significantly lower in HCV naive CD4 T cells in the 4-day culture compared to the HS, as measured by flow cytometry (Fig. 3d) and western blot (Fig. 3e). Similarly, the mRNA expressions of ATM signaling molecules, P53, 53BP1, BRCA1, CHK1, and CHK2 remained unchanged or even higher in naive CD4 T cells from HCV patients when compared to the HS (Fig. 3f). However, pp53 protein was undetectable in resting naive CD4 T cells without stimulation, whereas BRCA1 and CHK1 total and phosphorylated proteins remained unchanged (Fig. 3g); CHK2 protein, especially pCHK2, was significantly suppressed in HCV T cells compared to the HS, as demonstrated by western blot and flow cytometry (Fig. 3h). These results suggest that the DNA damage sensing machinery may be intact, but the DNA repair (ATM/CHK2) pathway is inhibited, at the post-transcriptional levels, especially the phosphorylation process essential for its activation and function.

### Role of HCV in dampening ATM activation and its effects on DNA damage and cell apoptosis

**Fig. 4:**
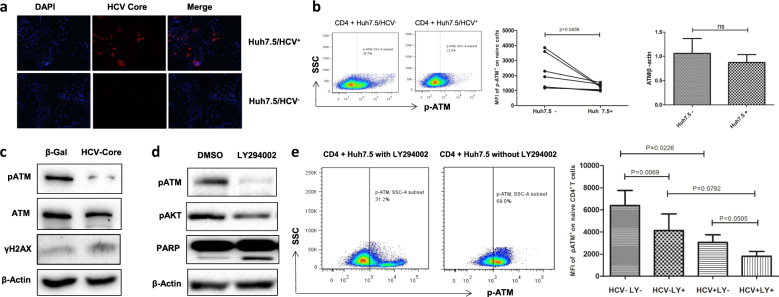
HCV inhibits ATM/pATM expressions via dampening PI3K pathway. **a** HCV infection of Huh7.5 hepatocytes. Immunostaining of HCV core protein is positive in HCV-infected cells, but negative in HCV-uninfected cells. **b** ATM/pATM is inhibited in naive CD4 T cells co-cultured with Huh7.5 cells with HCV infection compared with those incubated with Huh7.5 cells without HCV infection. **c** HCV core protein inhibits ATM/pATM and enhances γH2AX expressions in naive CD4 T cells compared with the β-Gal control. **d** PI3K inhibitor (LY294002) inhibits pATM/pAKT and enhances PARP expressions in naive CD4 T cells compared with those treated with DMSO control. **e** PI3K inhibitor (LY294002) inhibits pATM expressions in naive CD4 T cells co-cultured with HCV^+/−^ Huh7.5 cells compared with the cells without inhibitor treatment. Representative flow cytometry dot plots and summary data (*n*=8) with *P* value between the groups are shown

As chronically HCV-infected patients often have other co-morbidities that may cause immune dysregulation, we examined the specific role of HCV in triggering DDR and promoting cellular senescence or apoptosis. We incubated naive CD4 T cells with Huh7.5 cells with or without HCV infection, followed by measuring ATM activation. As shown in Fig. 4a (immunofluorescence staining of Huh7.5 cells with or without infection by HCV JFH-1 strain), hepatocytes transfected with HCV RNA at 48 h showed positive expression of HCV core protein, whereas cells with mock transfection exhibited negative staining. Moreover, we detected HCV RNA in the supernatant of HCV-transfected cells (at 24 h as well as 48 h), but not mock-transfected cells (data not shown). Importantly, similar to the observations in HCV patients (Fig. 3b), whereas the total ATM protein expression was not significantly decreased, the phosphorylation of ATM was markedly inhibited in the naive CD4 T cells that were incubated with HCV^+^ Huh7.5 cells compared with cells co-cultured with HCV^−^ Huh7.5 hepatocytes (Fig. 4b). In addition, T cells exposed to HCV-expressing hepatocytes were more apoptotic, as demonstrated by a significant increase (*P* = 0.0357) in Av/7AAD expression in CD4 T cells exposed to HCV compared with the negative control (data not shown).

We have previously shown that primary T cells treated with HCV core protein exhibited a senescent state, as evidenced by a higher level of the aging marker β-Galactosidase expression and shortened telomeres^5^. To further investigate whether HCV core-treated T cells have impairment in the DNA damage repair enzyme ATM, we exposed healthy naive CD4 T cells to HCV core protein, which is secreted by virally infected hepatocytes and circulates in the peripheral blood of HCV-infected patients and can dampen T cells through its interaction with the globulin head of C1q receptor expressed on the surface of T cells, thus delivering inhibitory signaling^29, 30^. As shown in Fig. 4c, compared to the β-gal control, treatment with HCV core protein for 5 days significantly inhibited the phosphorylation of ATM, but not total ATM protein expression, in naive CD4 T cells. In addition, HCV core-treated T cells exhibited an increased level of γH2AX (a marker of DNA damage), suggesting that insufficient pATM is associated with an increased DNA damage in HCV core-treated T cells.

ATM belongs to the PI3K family, and we have previously shown that HCV can induce T-cell senescence by inhibiting the AKT/PI3K pathway^3^. To further explore the mechanisms that might be involved in inhibiting ATM activation, we treated naive CD4 T cells with LY294002 (a potent PI3K-specific inhibitor) in the presence or absence of HCV core protein. As shown in Fig. 4d, compared with the DMSO-treated control, T cells treated with 20 μM LY294002 exhibited remarkable inhibition of pATM, along with decreases in pAKT. Moreover, the cleaved form of Poly (ADP-ribose) polymerase (PARP), an enzyme that catalyzes the transfer of ADP-ribose onto target proteins and plays an important role in DNA repair and cell survival, was significantly increased in T cells treated with the PI3K inhibitor. In addition, we assessed the effect of LY294002 on ATM inhibition in CD4 T cells co-cultured with HCV^+/−^ Huh7.5 cells. As shown in Fig. 4e, pATM expression in CD4 T cells in the presence of Huh7.5 hepatocytes without HCV infection was significantly inhibited by the PI3K inhibitor. pATM expression was further inhibited in naive CD4 T cells incubated with HCV^+^ Huh7.5 cells, especially in the presence of PI3K inhibitor. These results suggest that HCV (core protein) can induce T-cell DNA damage that is associated with impaired ATM via inhibition of the PI3K.

### Inhibition of ATM phosphorylation in naive CD4 T-cell leads to DNA damage and apoptosis

**Fig. 5:**
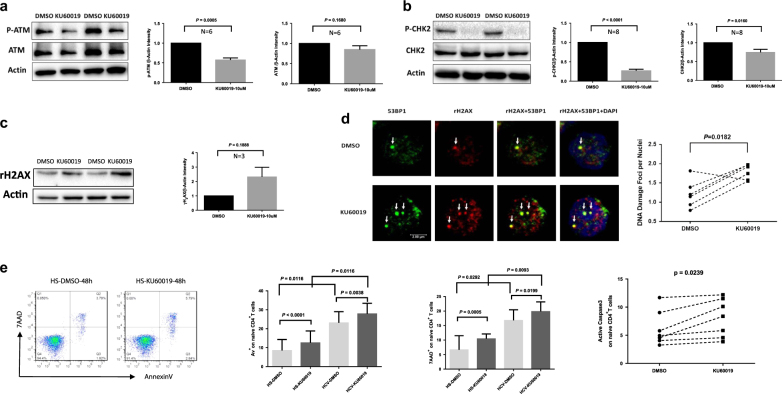
KU60019, an ATM inhibitor, induces DNA damage and cell apoptosis via inhibiting ATM pathway. **a** KU60019 inhibits ATM/pATM expressions in naive CD4 T cells. Representative imaging and summary data show western blot analysis for ATM/pATM expressions in naive CD4 T cells treated with 10 μM KU60019 or DMSO for 48 h. **b** KU60019 inhibits CHK2/pCHK2 expressions in naive CD4 T cells. **c** KU60019 enhances γH2AX expression in naive CD4 T cells. **d** KU60019 increases DNA damage foci (γH2AX/53BP1 co-localization) in naive CD4 T cells. **e** KU60019 increases T-cell apoptosis in naive CD4 T cells. Av, 7AAD, and active caspase-3 expressions are shown in naive CD4 T cells treated with KU60019 vs. DMSO control at 48 h

The ATM signaling pathway is pivotal to the maintenance of genome integrity and cell survival^26^. To test the functionality of this DNA repair machinery in T-cell survival, freshly isolated healthy naive (CD4^+^CD45RO^−^) T cells were treated with a specific ATM inhibitor (KU60019, 10 μM) for 48 h, followed by measuring DNA damage and cell apoptosis or death. As shown in Fig. 5a, naive T cells exposed to KU60019 showed an inhibition in ATM, in particular pATM, compared with the DMSO control. In addition, the phosphorylation of CHK2 protein, a downstream effector of ATM signaling pathway, was significantly inhibited by the treatment (Fig. 5b). Importantly, T cells exposed to the ATM inhibitor exhibited an elevated γH2AX expression, suggesting an increase in DNA damage (Fig. 5c). In addition, T cells treated with the ATM inhibitor exhibited an increase in DNA damage foci (γH2AX/53BP1 co-localization) compared with the control (Fig. 5d). Moreover, ATM inhibition in naive CD4 T cells resulted in considerately increased T-cell apoptosis and death, as demonstrated by an increase in Av and 7ADD expression, as well as activated caspase-3 following the treatment (Fig. 5e). In essence, an insufficiency of ATM leads to greater DNA damage and cell apoptotic death, which may necessitate compensatory homeostatic proliferation and lead to telomere loss and premature senescence, particularly in the naive T-cell pool, a mechanism that can potentially contribute to the naive T-cell loss and poor immune (vaccine) responses in chronic HCV infection.

### Reconstitution of ATM in HCV CD4 T cells repairs DNA damage, survival defect, and cellular functions

**Fig. 6:**
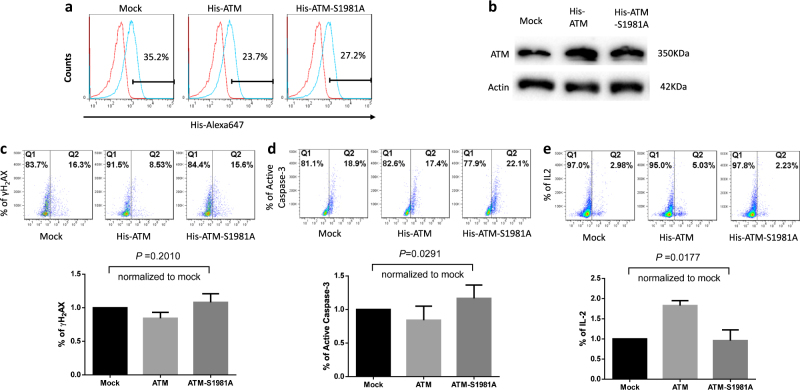
Ectopic ATM expression repairs DNA damage, cell apoptosis, and cellular dysfunctions from HCV infection. **a** Representative histogram of His Expression (Blue) in Mock-, ATM, and ATM-S1981A-transfected naive CD4 T cells derived from HCV-infected patients. Isotype control (Red) was used for gating the His^+^ cells in flow cytometry analysis. **b** ATM expression in Mock, ATM, and ATM-S1981A-transfected naive CD4 T cells from HCV-infected patients, measured by western blot. **c**, **d**, **e** Representative dot plots and summary data of flow cytometric analysis for the expression of γH2AX (*n*=10), active caspase-3 (*n*=9), and IL-2 (*n*=3) in Mock, ATM, and ATM-S1981A-transfected naive CD4 T cells derived from HCV-infected patients

Given the critical role of ATM in repairing DNA-DSBs, we hypothesized that reconstitution of ATM could protect HCV-derived T cells from DNA damage and restore the signaling network required for repairing DNA breaks. To test this, we transfected CD4 T cells derived from HCV-infected individuals with Flag-His-ATM constructs or controls, including an empty vector without ATM insert (Mock), and an ATM mutant (ATM-S1981A) in which the serine (S) phosphorylation site at residue number 1981 was substituted by alanine (A), using the Lonza transfection system. Despite high transfection efficiency (70%) with GFP transfection by this system, fluorescence-activated cell sorting analysis revealed intracellular His-ATM expression in only 20–40% of the T cells transfected with Flag-His-ATM or control constructs (Fig. 6a). Western blotting confirmed an increase in ATM expression in the wild-type, as well as mutant ATM-S1981A-transfected T cells (Fig. 6b). Although ectopic overexpression of ATM had broad biological consequences, we focused our investigation on the DNA repair, cell survival, and cell function, by assessing γH2AX, caspase-3, and IL-2 expression levels as readout. As shown in Fig. 6c, compared to mock transfection, ATM reconstitution reduced γH2AX expression after 48 h, whereas transfection of the non-phosphorylated form with an ATM-S1981A mutant was unable to restore the level of DNA repair, indicating the importance of ATM phosphorylation at serine 1981 in protecting cells from excessive DNA breaks. In parallel, transfection of wild-type ATM significantly reduced T-cell apoptosis, whereas mock- or ATM-S1981A-transfected cells exhibited relatively higher levels of active caspase-3 expression (Fig. 6d), suggesting that ectopic ATM expression exerts immediate effects in determining T-cell fate by securing cell survival. Most importantly, implementing ATM significantly improved T-cell function, as shown by the increase in IL-2 expression in ATM-transfected cells, whereas ATM-S1981A transfection could not induce such an effect (Fig. 6e). Taken together, these results suggest that restoring an adequate ATM level in naive CD4 T cells from chronic HCV infection is sufficient to ameliorate DNA damage, survival defects, and cell dysfunctions.

## Discussion

**Fig. 7:**
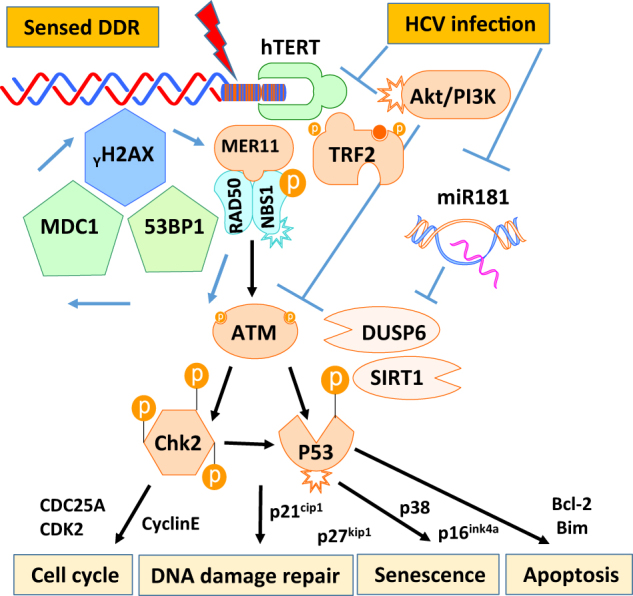
**A** **novel model of HCV-induced ATM deficiency in T-cell cycle arrest, DNA damage repair, cell senescence, and apoptosis.** HCV infection triggers a DNA damage response (DDR) in the early phase via activation of MRN-ATM-CHK2 and P53 signaling pathways in naive CD4 T cells, prompting cell cycle arrest and allowing for DNA damage repair; or, if the infection is overwhelming and causes unrepairable DNA damage, the cell will commit suicide and initiates programmed cell death (apoptosis). Persistent antigenic and inflammatory stimulation, however, drives ATM exhaustion and insufficiency, leading to impaired DNA damage repair and accumulation of DNA double strain breaks (DSBs), which result in constant cell apoptosis and naive T-cell loss. Excessive T-cell loss necessitates high homeostatic proliferation and imposes replicative stress on unprimed naive T cells, emerging as a novel molecular mechanism underlying T-cell senescence in the setting of chronic viral infection. Importantly, ectopic overexpression of ATM is necessary and sufficient to repair the DNA damage, survival deficit, and cellular dysfunction in HCV-derived T cells, providing a new strategy to improve immunotherapy and vaccine responses against human viral diseases

We and others have previously shown that T cells derived from patients with chronic viral infections prematurely reach senescence, characterized by the shortening of telomeres and expression of aging markers^2–6^. In this study, we further demonstrate that homeostatic remodeling of the T-cell repertoire during HCV infection primarily affects the naive T-cell compartment, characterized by an accumulation of DNA damage owing to insufficient activation of the DNA repair ATM enzyme, which leads to naive T-cell apoptosis and loss. Indeed, phosphorylation of ATM in T cells exposed to HCV (core protein) is inhibited through dampening the PI3K pathway, resulting in an increase in DNA damage and cell apoptosis. Moreover, pharmacological inhibition of ATM phosphorylation leads to more DNA damage and apoptosis in naive T cells. Most importantly, reconstitution of ATM repairs the DNA damage, cell apoptosis, and functional defects in naive CD4 T cells derived from HCV-infected patients. Based on these novel findings, we propose a model (depicted in Fig. 7) where HCV-induced ATM deficiency leads to accumulation of DNA damage and cell apoptosis. The excessive T-cell loss necessitates high homeostatic proliferation and imposes replicative stress on unprimed naive T cells; this represents a novel molecular mechanism underlying T-cell senescence in the setting of chronic viral infection. Importantly, ectopic overexpression of ATM is necessary and sufficient to repair the DNA damage, survival defect, and cell dysfunctions in HCV-derived T cells, thus providing a new strategy to improve immunotherapy and vaccine responses against human viral diseases.

We and others have observed poor vaccine responses in the setting of chronic viral (HCV, HIV) infections^2, 3, 15–19^, but the underlying mechanisms for vaccine failure in virally infected individuals remain unclear. Data presented in this study indicate that naive helper T cells in chronically HCV-infected patients have abnormalities that jeopardize their ability to mount effective immune (vaccine) responses. Specifically, we demonstrated that naive CD4 T cells have accumulation of damaged DNA and fail to repair their DNA-DSBs owing to deficiency of the ATM pathway. Accumulated DNA damage renders HCV-derived T cells prone to apoptotic death, imposing replicative stress and premature aging on naive T cells. These findings are important because naive T cells represent the reserve pool of the immune system, and their survival critically determines the cellular yield of homeostatic proliferation, a process that generates new T cells in response to neo-antigens, including vaccines.

Insufficient activation of ATM would be expected to affect both unprimed and primed T cells. Indeed, we observed unrepaired DNA damage and cell apoptosis in both naive and memory T-cell populations. However, ongoing antigenic stimulation during chronic viral infection could drive naive T-cell differentiation and turnover of antigen-reactive T cells. In this regard, memory T cells would expand and compromise the size and survival of naive T cells. Eventually, the entire T-cell pool would be comprised by antigen-reactive T cells at the expense of naive T cells. In the setting of chronic viral infection, however, memory T cells could be functionally biased as a result of chronic antigenic stimulation; as such, we focused our studies on the T-cell population that has yet to be recruited for immune responses. With the decrease in newly generated naive thymic T cells in adults, chronic infection or inflammation might force the immune system to restore equilibrium by replicating the available or existing naive T cells, thereby driving telomere shortening and senescence in naive T-cell populations. Thus, the ability to generate immune response to new antigens, such as HBV vaccine, could be compromised.

ATM has a unique role in lymphocyte biology, as programmed DNA damage repair is part of the gene rearrangement necessary for formation of a highly diverse T-cell receptor repertoire. Under non-stress conditions, ATM is inactive and exists in the form of a dimer (like other PI3Ks). It requires a signal for activation (usually a DNA damage signal), signaling through intermolecular autophosphorylation at ATM residue S1981 and resulting in the dimer dissociation into monomers^31^. In our study, a mutant of S1981A rendered ATM a dominant-negative protein, triggering more severe DNA damage (γH2AX expression), cell apoptotic death (Av/7AAD expression), and cellular dysfunction (IL-2 inhibition)—underscoring the importance of ATM phosphorylation for its biological functions. ATM is predominantly localized in the nucleus, and undergoes activation once the MRN complex senses and binds to DNA DSB ends, providing a platform for ATM recruitment and autophosphorylation^32, 33^. Phosphorylation of S1981 also stabilizes ATM at the damaged DNA sites and recruits more downstream effector proteins to participate in the DDR^34^. Among the multiple substrates phosphorylated by ATM is the checkpoint kinase 2 (CHK2), which is phosphorylated at residue T62 following DSB formation and prevents cells from progressing from G1 to S phase or, alternatively, leads to cell apoptosis. The discovery of these DNA damage response proteins has shed light on the cellular machinery that contributes to DNA repair and cell homeostasis.

Recently, Li et al.^35^ identified prematurely aged T cells with damaged telomeres in patients with rheumatoid arthritis, resulting from defective activity of the DNA break sensor MER11A. In patients with chronic HCV infection, however, we find that the DNA damage sensor MRN complex is intact in naive T cells. Rather, the DNA damage repair enzyme ATM is inhibited, at a post-transcriptional level, by HCV infection. Interestingly, Guo et al.^36, 37^ reported that ATM activation in response to ROS was independent of the MRN complex. ROS-mediated ATM signaling represses mTORC1 signaling and therefore cell growth and proliferation through activation of TSC2 (a negative regulator of mTOR) by liver kinase B1 (also known as STK11) and AMP-dependent protein kinases^38^. ATM engagement of the TSC2/mTORC1 signaling pathway can also regulate autophagy^39^, and differential localization of ATM is correlated with activation of distinct downstream signaling pathways^40^. We have previously reported that T cells treated with HCV core protein exhibit G1/S cell cycle arrest, which was associated with the dysregulation of cell cycle regulatory proteins CDKs/Cyclins and P27^kip1^ ^41^. Here, we demonstrate that concomitant with the insufficiency of ATM activation, the phosphorylation of CHK2 is defective in naive T cells from HCV-infected patients. Moreover, pharmacological inhibition of ATM in healthy T cells also leads to a CHK2 defect, accompanied by a marked increase in DNA damage and cell apoptosis—resembling the biological effects characteristic of HCV-derived naive T cells. These results establish that in human T cells, CHK2 is targeted by ATM, and that the overall defect of this pathway can be attributed to ATM insufficiency owing to chronic HCV infection.

A typical feature of CD4 T cells in chronically HCV-infected patients is the shortening of telomeres compared with age-matched healthy controls^5, 6^. Several mechanisms may contribute to this age-associated loss of telomeres. Increased proliferative turnover can cause cell division-induced telomere shortening. In addition, telomeric DNA is highly susceptible to DNA damage, even more so than non-telomeric DNA. Plasmid-inserted human telomeres accumulate sevenfold higher strand breakage than control sequences^42^. Also, the frequency of single-strand breaks is several-fold higher in telomeres than in the bulk genome when cells are treated with alkylating agents or exposed to oxidative stress^43^. In line with these findings, we have recently found that the expression of the telomere shelterin TRF2 is significantly inhibited, at the protein level, in naive CD4 T cells derived from HCV-infected individuals, which renders the uncapped telomeres prone to DNA damage (unpublished observations). Thus, telomere loss in HCV T cells is triggered by DDR and the inability of timely repair by the ATM signaling pathway. In addition, we have also discovered that KML001, a telomere-targeting drug, can induce telomeric DNA damage and T-cell apoptosis by impairing the ATM pathway (unpublished observations). Notably, ATM is widely expressed in human T cells at an extremely high level to ensure integrity of the genomic DNA in replicating lymphocytes. ATM activation represents the initiation of DDR, but its inhibition in persistently stimulated T cells indicates insufficiency of this DNA repair enzyme and cell exhaustion and senescence in the setting of chronic viral infection. This notion is supported by our observation in an in vitro stimulated T-cell system that ATM phosphorylation is increased in the early phase of KML001-treated T cells (3~6 h) and decreased in persistently treated cells (24~48 h), along with increases in DNA damage, cell apoptosis, and functional impairment (unpublished observations).

In summary, accumulation of DNA damage and failure to repair the DNA-DSBs owing to deficiency of the ATM-dependent DNA repair machinery during chronic viral infection may have broader implications through impairing diverse cellular functions. As interferon (IFN)-mediated T-cell apoptotic death has been well-studied in persistent viral infections^44–47^, this virus-induced DNA damage-mediated T-cell loss represents a new mechanism of immune evasion. How HCV induces DNA-DSBs, and its relationship to the IFN-signaling pathway, are under further investigation. As counteracting ATM deficiency may restore T-cell competency during viral infection and prevent premature immune aging, these studies may provide new strategies to improve immunotherapy and vaccine responses against human viral diseases.

## Materials and Methods

### Subjects

The study protocol was approved by the institutional review board (IRB) of East Tennessee State University and James H. Quillen VA Medical Center (ETSU/VA IRB, Johnson City, TN, USA). The study subjects were composed of two populations: 148 chronically HCV-infected individuals and 72 age-matched HS. Written informed consent was obtained from all participants. HCV patients were virologically positive for HCV RNA, prior to the antiviral treatment. Healthy subjects, derived from Physicians Plasma Alliance, Gray, TN, USA were negative for HBV, HCV, and HIV infection.

### Cell isolation and culture

PBMCs were isolated from whole blood by Ficoll (GE Heathcare, Piscataway, NJ, USA) density centrifugation. Naive and memory CD4^+^ T cells were isolated from PBMCs using the naive or Memory CD4^+^ T Cell Isolation Kit and a MidiMACS Separator (Miltenyi Biotec Inc., Auburn, CA). The isolated T cells were cultured in RPMI 1640 medium containing 10% FBS (Atlanta Biologicals, Flowery Branch, GA, USA), 100 IU/ml penicillin and 2 mM L-glutamine (Thermo Scientific, Logan, UT, USA) without mitogenic stimulation for 4 days at 37 °C and 5% CO2 atmosphere. Cells were collected at day 0, day 2, or day 4 for detection of cell apoptosis and DNA damage. To test the role of pATM in repairing DNA damage and apoptosis, 10 μM pATM inhibitor (KU60019, Abcam, Cambridge, MA) or dimethyl sulphoxide were added to the cultures for 48 h, followed by apoptosis and DNA damage analysis. To consolidate the role of HCV in inhibiting ATM activation, purified naive CD4 T cells were co-cultured with Huh7.5 cells with or without HCV infection, or 1 µg/ml recombinant HCV core protein (Virogen, watertown, MA, USA) or control protein β-galactosidase (Virogen), in the presence or absence of 20 μM PI3K inhibitor (LY294002, Sigma) for 4 days, followed by flow cytometry or Western blot analysis for ATM/pATM expression, DNA damage, and cell apoptosis.

### Flow cytometry

For phenotypic analysis of naive CD4 T cells, PBMCs were stained with CD4-APC, CD45RA-FITC (BioLegend, San Diego, CA, USA) antibodies, or isotype controls. To quantify cell apoptosis, naive or memory cells were purified, cultured, and collected at indicated days and stained with Av and 7AAD using BD Pharmingen PE Av Apoptosis Detection Kit I (BD Biosciences, San Jose, CA, USA). For intracellular staining, the cells were fixed and permeabilized with Foxp3 Transcription Factor Staining Buffer Set (eBioscience, San Diego, CA, USA), and stained with pATM (Ser1981)-PE antibody(BioLegend), ATM antibody (Abcam), and anti-Rabbit-IgG-Alexa Fluor 488 (Santa Cruz Biotechnology, Dallas, TX, USA), pCHK2 (Thr68)-PE antibody (eBioscience), 8-oxoguaine-FITC probe (OxyDNA Assay Kit, EMD Millipore, Billerica, MA). The stained cells were analyzed on AccuriTM C6 flow cytometer (BD, Franklin Lakes, NJ), and data were analyzed by FlowJo software (Tree Star, Inc., Ashland, OR). Isotype control antibodies (eBioscience) and fluorescence minus one controls were used to determine the background levels of staining and adjust multicolor compensation as gating strategy.

### RNA isolation and real-time RT-PCR

Total RNA was extracted from 1.0 × 10^6^ cells with PureLink RNA Mini Kit (Invitrogen, Carlsbad, CA), and cDNA was synthesized by using High Capacity cDNA Reverse Transcription Kit (Applied Bio systems, Foster city, CA) per manufacturer’s instruction. Quantitative PCR were completed in triplicates following the conditions 95 °C, 10 min and then 95 °C, 15 s; 60 °C, 60 s with 40 cycles. Gene expression was normalized to 18S ribosomal RNA and expressed as fold changes using the 2^−ΔΔct^ method. Primer sequences were shown in Table 1.

**Table 1:** Primer sequences

Name of the amplified genes	Primer sequences
ATM	F: 5′-TGGATCCAGCTATTTGGTTTGA-3′
	R: 5′−GATGAAGAAGATAACAACCAATGTATGAACC−3′
p53	F: 5′−TCAACAAGATGTTTTGCCAACTG−3′
	R: 5′−GTAGATGTTCGTCAGTGTCGTGTA−3′
MRE11	F: 5′−CTTGTACGACTGCGAGTGGA−3′
	R: 5′−GTCTTTCTCCCTACCCACTT−3′
NBS1	F: 5′−TTGGTTGCATGCTCTTCTTG−3′
	R: 5′−CAACTCAGGTTCTTCGTCGG−3′
RAD50	F: 5′−CTTGGATATGCGAGGACGAT−3′
	R: 5′−CGCATTGAAGGTCGAAGACC−3′
BRCA1	F: 5′−GGCTATCCTCTCAGAGTGACA−3′
	R: 5′−AGGTCTTGTTTCGTGTAGTC−3′
CHEK1	F: 5′−GGTGAATATAGTGCTGCTATGTTGACA−3′
	R: 5′−CACAGTGAAGGGACAAATAGGTT−3′
CHEK2	F: 5′−CCCAAGGCTCCTCCTCACA−3′
	R: 5′−TTGAGGTCGGTCAGGAGAGTGA−3′
18S–ribosomal RNA	F: 5′−CCTGGATACCGCAGCTAGGA−3′
	R: 5′−CCCCGTAAGCATAACGCGGCG−3′

### Western blotting

Naive CD4 T cells purified from HCV-infected individuals and HS were lysed on ice in RIPA lysis buffer (Boston BioProducts Inc, Ashland, MA) in the presence of protease inhibitors (Thermo Scientific, Rockford, IL). The protein concentrations were measured by Pierce BCA protein assay kit (Thermo Scientific). Proteins were separated by SDS-PAGE, transferred to polyvinylidene difluoride membranes, which were blocked with 5% non-fat milk, 0.5% Tween-20 in Tris buffered saline, and incubated with the pATM (Ser1981) (D6H9), pBRCA1, pCHK1, and pCHK2 (Thr68) (C13C1) antibodies and β-Actin (8H10D10) antibodies (Cell Signaling, Danvers, MA). Appropriate horseradish peroxide-conjugated secondary antibodies (Cell Signaling) was then used and proteins were detected using Amersham ECL Prime Western Blotting Detection Reagent (GE Healthcare Bio-Sciences, Pittsburgh, PA). Membranes were stripped and re-probed with MER11, RAD50, NBS1, BRCA1, ATM (D2E2), γH2AX, PARP, CHK1, and CHK2 (D9C6) antibodies (Cell Signaling). Protein bands were captured and quantitatively analyzed by Chemi DocTM MP Imaging System (Bio-Rad System).

### Confocal microscopy

Naive CD4^+^ T cells were isolated and cultured as described above. Immunofluorescence staining was performed according to the reported method^39^. In brief, the cells were fixed in 2% paraformaldehyde for 20 min, permeabilized with 0.3% Triton X-100 in PBS for 10 min, blocked with 5% BSA in PBS for 1 h, and then incubated with rabbit anti-53BP1 antibody (Cell Signaling) and mouse anti-γ-H_2_AX (Ser-139) antibody (Biolegend) at 4 °C overnight. The cells were washed with PBS with 0.1% Tween-20 for three times, and then stained with anti-rabbit IgG-Alexa Fluor 488 and anti-mouse IgG- Alexa Fluor 555 (Invitrogen) at room temperature for 1 h, washed and mounted with DAPI Fluoromount-G (SouthernBiotech, Birmingham, AL). Images were acquired with a confocal laser-scanning inverted microscope (Leica Confocal, Model TCS sp8, Germany).

### ATM transfection

Purified naive CD4^+^ T cells from HCV patients were transfected with 2.5 µg pcDNA3.1 (a gift from Adam Antebi^48^, Addgene plasmid # 52534), or pcDNA3.1(+)Flag-His-ATM wt (a gift from Michael Kastan^49^, Addgene plasmid # 31985), or hATMS1981A mutant (a gift from Michael Kastan^31^, Addgene plasmid # 32300), using the Human T Nucelofector Kit and Nucleofector I Device (LonzaLonza, Allendale, NJ). 24 h post transfection, GFP fluorescence was observed under microscope and the GFP expression level was measured by Flow Cytometry. 48 h post transfection, transfection efficiencies were monitored by flow cytometry measuring the frequency of His^+^ cells. Ectopic ATM expression was detected by western blot. Active caspase-3, γH_2_AX, and IL-2 expressions were assessed in the His-positive cells by Flow Cytometry.

### Statistical analysis

The data were summarized as mean±SEM or median with interquartile range and analyzed using Prism 7 software. Comparisons between two groups were made using independent Student’s *t-*test, or paired *T* test, and multiple comparisons test/least significant difference or Tukey’s procedure, depending on the ANOVA *F* test or by a nonparametric Mann–Whitney *U*-test. *P*-values<0.05, <0.01, or <0.001 were considered to be statistically significant or very significant, respectively.

## Electronic supplementary material

Supplementary Figure S1

Supplementary Figure S1 legends
