# Supplementary material for: Insufficiency of DNA repair enzyme ATM promotes naive CD4 T-cell loss in chronic hepatitis C virus infection
Source: Cell Discov. 2018 Apr 10;4:16. doi: 10.1038/s41421-018-0015-4 (PMC5891503; doi:10.1038/s41421-018-0015-4)
Supplement: Supplementary file 1 — Supplementary Figure S1 [file 41421_2018_15_MOESM1_ESM.pptx]

## Slide 1
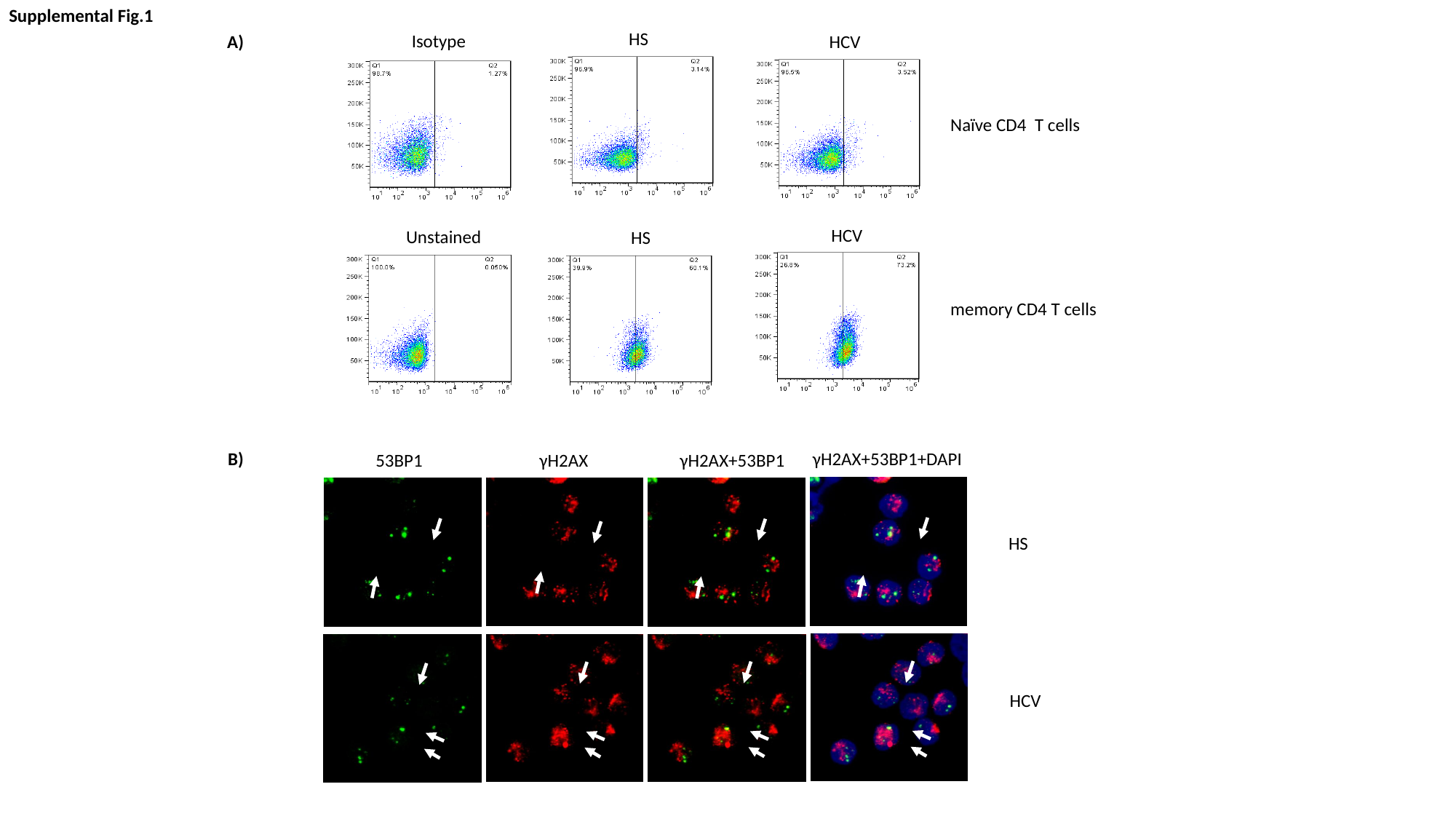

Supplemental Fig.1
HS
Isotype
A)
HCV
Naïve CD4 T cells
HCV
 Unstained
HS
memory CD4 T cells
B)
γH2AX+53BP1+DAPI
γH2AX
γH2AX+53BP1
53BP1
HS
HCV
