# Supplementary material for: Insufficiency of DNA repair enzyme ATM promotes naive CD4 T-cell loss in chronic hepatitis C virus infection
Source: Cell Discov. 2018 Apr 10;4:16. doi: 10.1038/s41421-018-0015-4 (PMC5891503; doi:10.1038/s41421-018-0015-4)
Supplement: Supplementary file 2 — Supplementary Figure S1 legends [file 41421_2018_15_MOESM2_ESM.docx]

**Supplementary Figure S1** **A)** Flow cytometric analysis of Fas receptor expression on resting naïve and memory CD4 T cells in PBMCs derived from HCV patients and HS. **B)** Confocal microscopic analysis of 53BP1/γH2AX co-localization in memory CD4 T cells isolated from HCV-infected patient and HS. Data were reproducible in cells derived from multiple subjects.
